# Dataset on spatial variability of soil properties: Tokhmeyevo archaeological site of the Bronze Age, Chuvashia (southern fringe of the forest zone, the Russian Plain)

**DOI:** 10.1016/j.dib.2020.106489

**Published:** 2020-11-04

**Authors:** Alexander Makeev, Alexey Rusakov, Olga Khokhlova, Pavel Kust, Daria Mikhaylova, Elena Aseyeva, Fatima Kurbanova, Elena Rusakova, Evgeniy Mihailov

**Affiliations:** a Soil Science Faculty, M.V. Lomonosov Moscow State University, Leninskie Gori, MSU, 1-12, 119991, Moscow, Russian Federation; b Saint Petersburg State University, 7-9, Universitetskaya nab., Saint Petersburg, 199034, Russian Federation; c Institute of Physicochemical and Biological Problems of Soil Science, Russian Academy of Sciences, Institutskaya street, 2, Pushchino, 142290, Russian Federation; d V.V. Dokuchaev Soil Science Institute, Russian Academy of Science, Pyzhevsky lane, 7, 109017, Moscow, Russian Federation; e Faculty of Geography, M.V. Lomonosov Moscow State University, Leninskie Gori, MSU, 1-12, 119991, Moscow, Russian Federation; f Chuvash State Institute of Humanitarian Sciences, Cheboksary, Moscow avenue, 29, Russian Federation

**Keywords:** Paleosols, Geoarchaeology, Retisol, Kurgan, Palynological analysis, Paleoenvironmental reconstruction, Holocene

## Abstract

Geoarchaeological and palaeopedological studies focusing on the reconstruction of the Holocene paleoenvironments require a detailed knowledge of the spatial variability of soil properties both for the surface soils and paleosols buried under archaeological constructions. However, such studies are often carried out at unique sites where it is difficult to ensure the representativeness of the data obtained. In this paper, we report original data on 15 soil profiles which shows the range of spatial variability of soil properties (рН H_2_O, рН KCl, particle size distribution, depth of genetic horizons, colour codes) for both surface and buried soils at the Tokhmeyevo kurgan cemetery, located in the Middle Volga region, Chuvash Republic, Russia. The data supplement the original research [1] and also give additional detailed information on pollen and spore analysis by plant species for the humus horizons in four buried and one surface soils. All soils developed from the same lithology (mantle loam), at the same elevation, in a similar topographic position (levelled upland slope) and in proximity to each other. Both buried and surface soils, classified as Retisols [1], show slight variability in morphology and particle size distribution that varies in a similar range. However, the two soil groups (buried and surface) differ in two striking features: buried soils exhibit dark humus horizon and black humic cutans in the middle part of the soil profile; these features are absent in the surface soils. The values of рН in water and 1 M KCl suspension in the buried soils and soils of the kurgan mounds are lower than in the surface soils. The data on the spatial variation of the properties of the surface and buried soils increase the reliability of the results, making it possible to assess the extent to which the differences in soils are associated with the environmental evolution. The presented data can provide one the context for further work in paleoenvironmental studies and also be compared with other already published datasets increasing the reliability of conclusions about the trends of environmental evolution in the second half of the Holocene.

## Specifications Table

**Table:** 

Subject	Environmental Science		
Specific subject area	Soil Science, Paleopedology, Soil Evolution, Geoarchaeology		
Type of data	Tables with raw data; Figures		
How data were acquired	Data on physicochemical properties of soils were obtained by means of standard techniques [2,3]		
Data format	Raw		
Parameters for data collection	Samples were collected from soil pits and boreholes within and just beyond the boundaries of the Tokhmeyevo archaeological site occupying a very gentle upland slope (Fig. 1). A major condition for the choice of the sampling locations was that the sampled pedons belong to a single landscape unit where all physiographic parameters (parent material, topography, vegetation) are similar and microtopographic features are not notable.		
Description of data collection	The Tokhmeyevo kurgan cemetery occupies an area of ∼6000 sq.m. Samples from the soil pits were collected from the soil horizons: Ah-AhE-E-E/Bt1 for the surface soils and Ahb-AhEb-Eb-EBtb-Bt1b for the buried soils. Borehole samples were taken at intervals of 10 cm down to a depth of 110 cm. The borehole samples from the burial mounds and the buried soils were taken from three kurgans through the burial mound and down to a depth of 100 cm from the former day surface; kurgan 4 was excavated by hand and sampled from the soil pit 4b. The samples from the surface soils were taken from seven soil pits deepened by boreholes. The earlier published data on soil properties of surface soil 1 s and the buried soil 8b and burial mound 8 [1] were included for comparison. The collected bulk samples were dried, sieved and used for physical fractionation into five particle size fractions and physicochemical analyses using conventional methods [2,3].		
Data source location	The sampling sites are located in the northern part of the Volga upland in 20 km west of Cheboksary, Cheboksary district, Chuvash Republic, Russia, 1 km south-west from Tokhmeevo village. The location of the sampling site on the Russian Plain is presented in [1]. GPS coordinates for the sampling locations were as follows:		
	1s	55.964320°N	47.165750°E
	3s	55.963840°N	47.166740°E
	4s	55.963207°N	47.167367°E
	5s	55.962444°N	47.168911°E
	6s	55.964409°N	47.163594°E
	7s	55.964053°N	47.162835°E
	8s	55.963313°N	47.161911°E
	9s	55.964063°N	47.163804°E
	4b	55.963793°N	47.165675°E
	5b	55.964103°N	47.166348°E
	6b	55.963803°N	47.166446°E
	7b	55.963990°N	47.166366°E
	8b	55.578480°N	47.988860°E
	9b	55.964065°N	47.166651°E
	24b	55.964140° N	47.164858°E
Data accessibility	Data are with this article		
Related research article	A. Makeev, A. Rusakov, F. Kurbanova, O. Khokhlova, P. Kust, M. Lebedeva, E. Milanovskiy, M. Egli, E. Denisova, E. Aseyeva, E. Rusakova, E. Mihailov. Soils at archaeological monuments of the Bronze Age – a key to the Holocene landscape dynamics in the broadleaf forest area of the Russian Plain. Quaternary International, In Press. https://doi.org/10.1016/j.quaint.2020.09.015.		

## Value of the Data

• The data show the importance of taking into account the spatial variability of soil properties for paleolandscape reconstructions based on the study of soil chronosequences. The dataset will contribute to a better understanding of the difference between spatial and temporal trends of the most labile (pH) and more conservative (grain size distribution) soil properties. Specifically, these data supported an important conclusion that due to the bioclimatic trend the black humus horizons and the black humic coatings degraded in the surface soils since the Bronze Age [1].
• The data can be used for improving the methodological approach in geoarchaeological research, highlighting the importance of spatial studies to increase the reliability of environmental reconstructions. They can be used by any researcher who undertakes a study of environmental trends based on paleosols properties.
• Detailed palynological data presented in this article can be used for independent paleoenvironmental reconstruction and will contribute to the pollen data bank.

## 1 Data Description

**Fig. 1:**
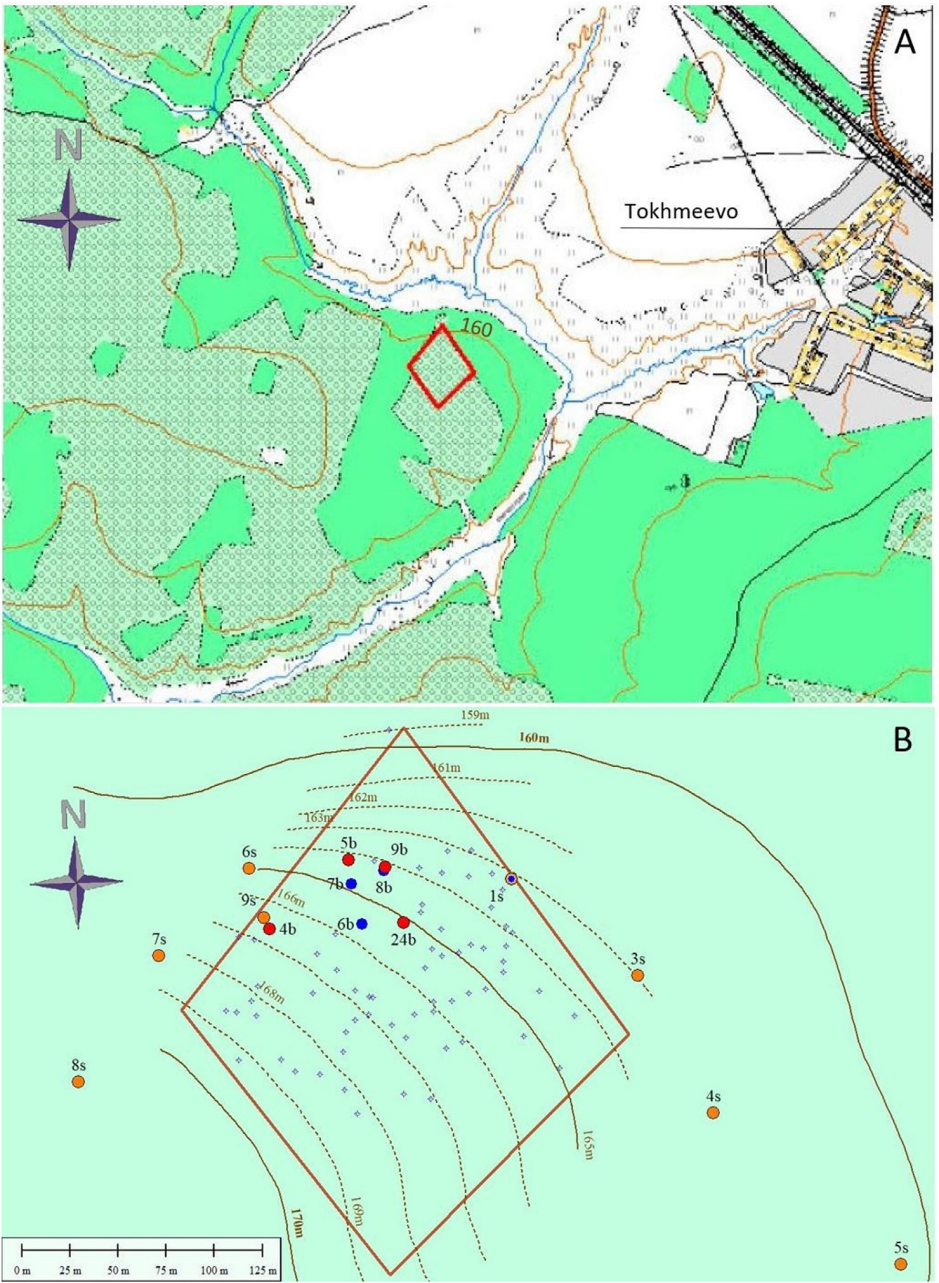
Topographic map of the study area (1:10,000, A), and the location of the studied soils (B). Red quadrangle marks the boundaries of archaeological monument (Tokhmeevo I kurgan cemetery). The soils investigated in 2020 are indicated as solid orange and red circles (the surface and the buried soils, respectively). Solid blue circles display the soils examined in 2018 [1] and small blue open stars show the location of unexplored kurgans. The buried soils, denoted with letter b, are labelled according to the number of kurgans [4]. Note that all surface soil profiles, denoted with letter s, are located in the northern part of the upland slope in the same topographic position as the kurgans.

Fig. 1 displays soil sampling locations, as well as topography and the boundaries of the archaeological site – the Tokhmeyevo kurgan cemetery. The cemetery is situated in a typical position for the Bronze Age kurgans: it occupies levelled and slightly sloping upland surface (160 – 170 m a.s.l.) at the edge of a small river valley side (Fig. 1A). The locations of the kurgans which are shown on Fig. 1B (both explored and unexplored) were obtained from [4]. It is well seen that the studied surface soils, which numbers are supplemented by the lowercase letter “s”, are located in the same topographic positions as the investigated kurgans (denoted with an official number and letter “b”; the latter means a soil pit or a borehole on a kurgan), but also characterize other parts of the slope.

**Fig. 2:**
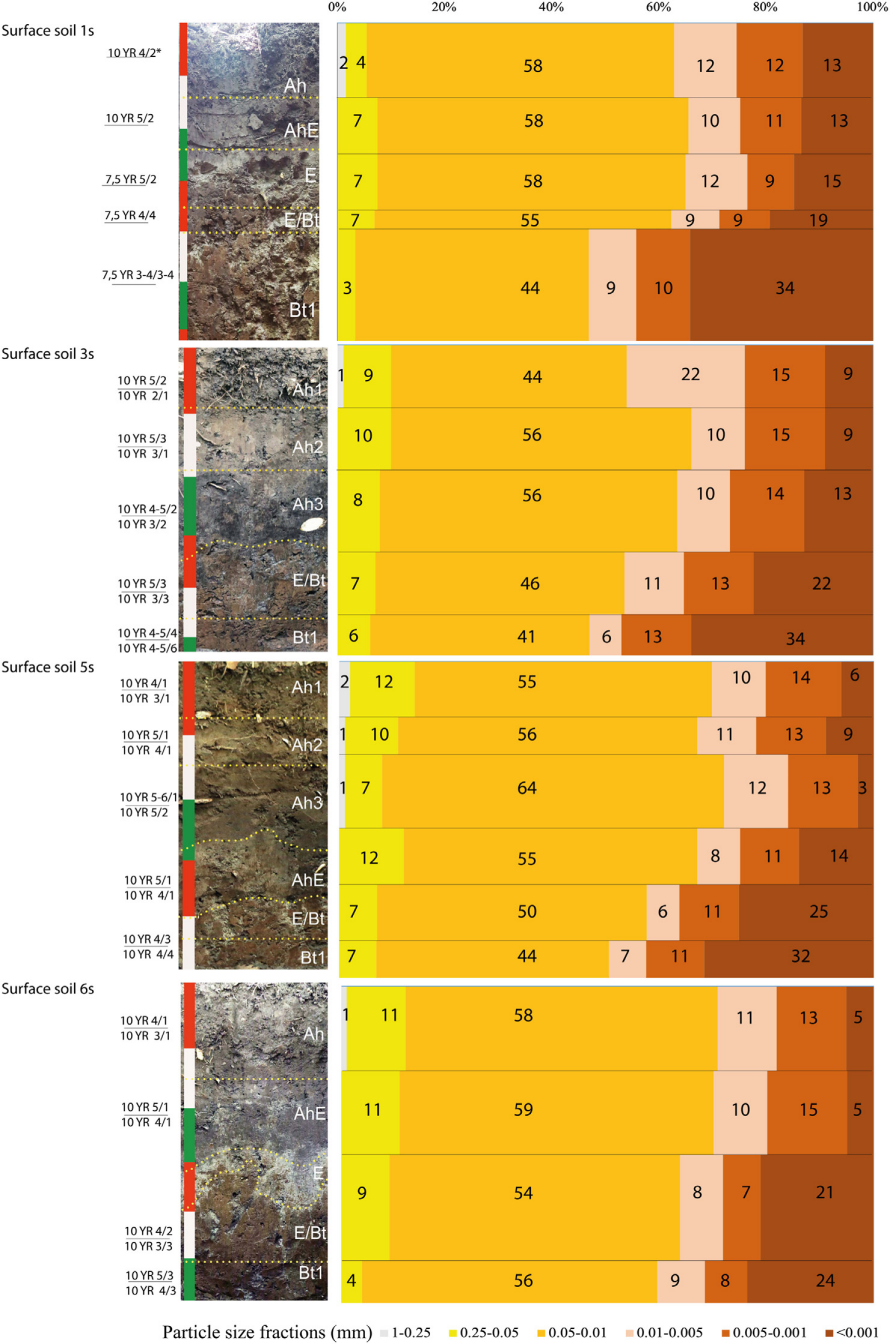

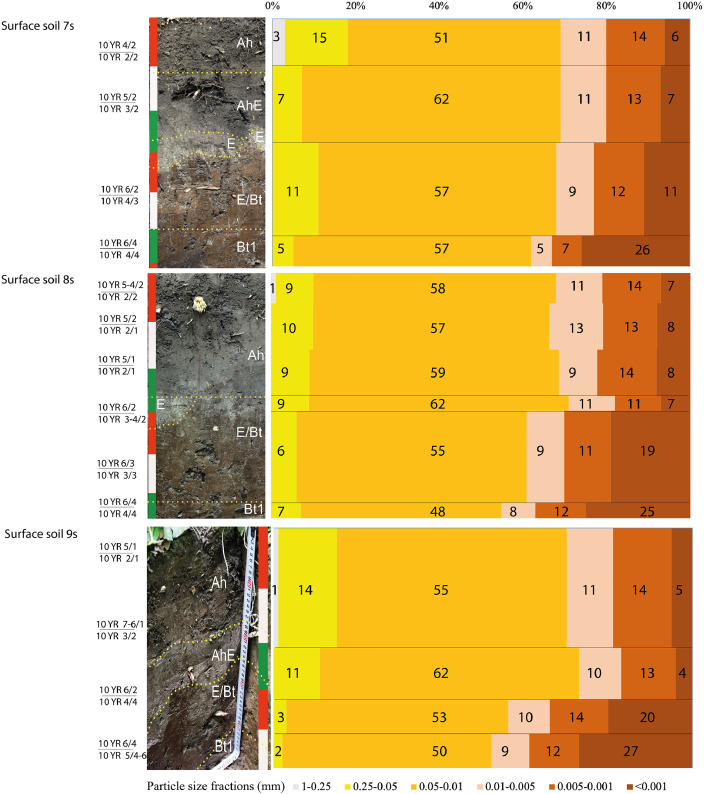
Field morphology and particle size distributions in the surface soils at the Tokhmeyevo kurgan cemetery. *Colours according to Munsell Soil colour Charts [7] (in numerator –when dry, in denominator – when moist).

**Fig. 3:**
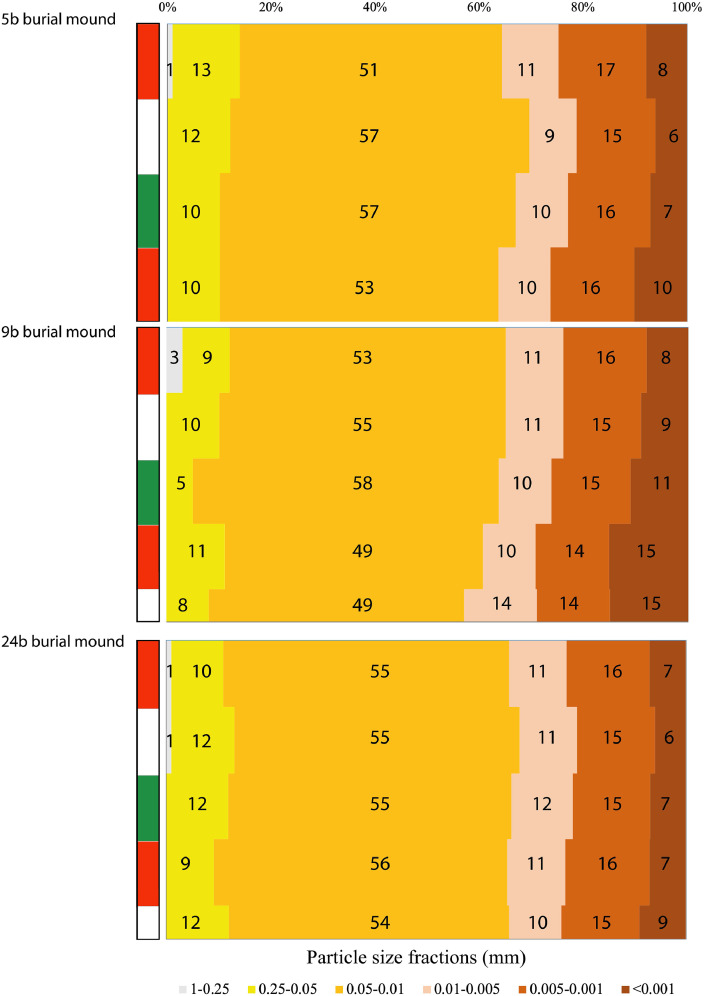
Particle size distribution in the soils of the burial mounds at the Tokhmeyevo kurgan cemetery.

Fig. 2 presents the basic features of field morphology of the surface soils in the seven locations (moist and dry Munsell colors, horizonation, photo images with photo tapes showing vertical metric scale) and particle size distribution indicating abrupt textural change typical for the soils of the area. Fig. 3 displays particle size distribution for the cover layer of the burial mounds (upper 40-45 cm) indicating that it is similar to that of the upper horizons of both surface and buried soils (see also [1]).

**Fig. 4:**
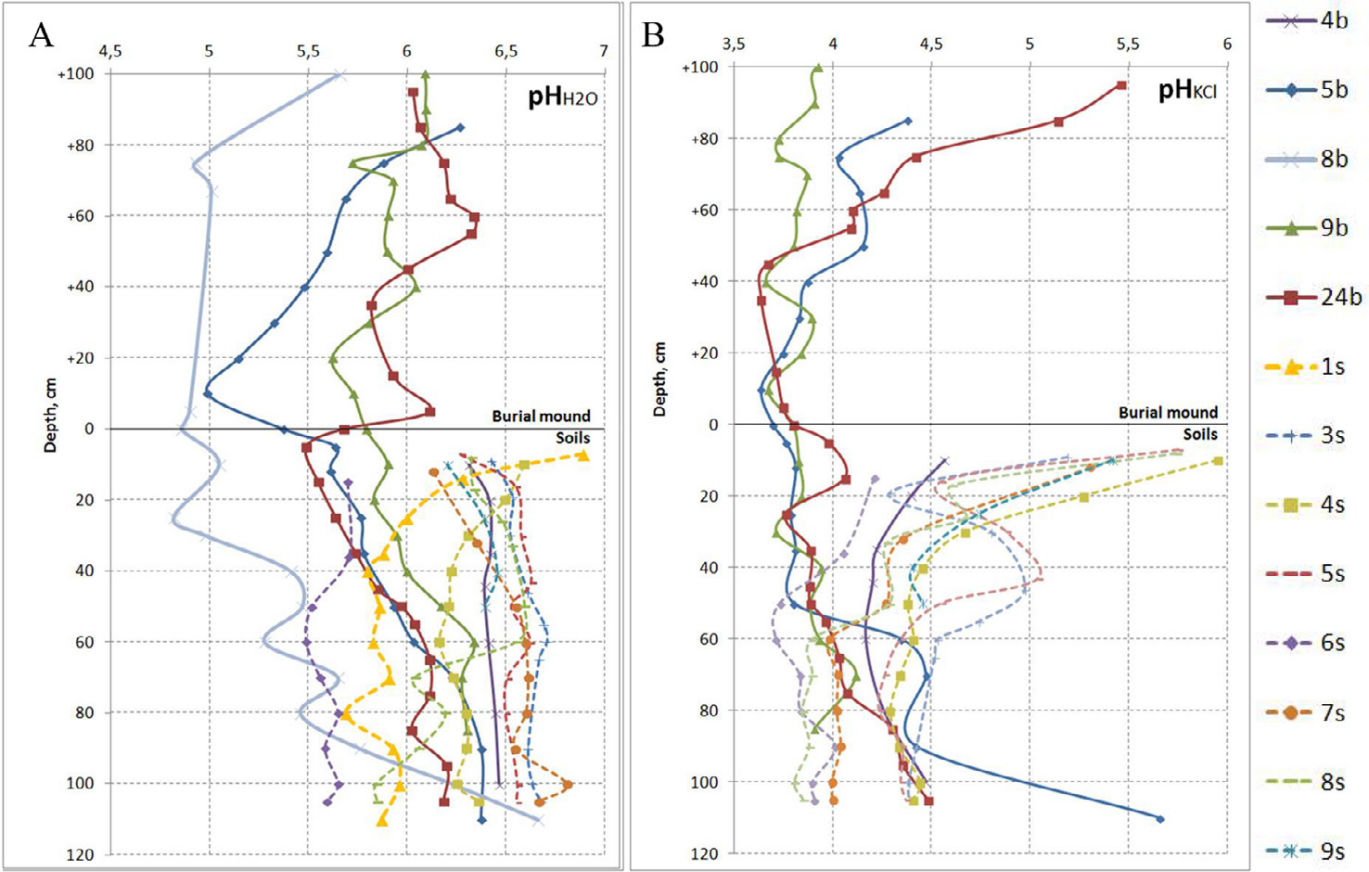
Depth distribution of pH values in the soils at the Tokhmeyevo kurgan cemetery. A – pH_H2O_; B – pH_KCl_. Solid lines – soils of the kurgans both of the burial mounds and ones buried under them; dashed lines – surface soils. Zero level – day surface for the buried and surface soils.

Table 1 provides raw data on рН in water and 1 М КСl suspensions of the surface and buried soils and the kurgan mounds. For visualizing, these data are also presented in the form of graphs (Fig. 4) showing that the upper horizons of the buried soils and the burial mounds are generally more acid than the surface soils. pH values for the separating layers in kurgans 8b and 9b are similar for those in the neighbouring horizons. Also in the buried soil 4b pH values are similar to those in the surface soils because the soil is now developing close to the day surface after archaeological excavation 40 years ago.

**Table 1:** pH values of the surface and buried soils and burial mounds at the Tokhmeyevo archaeological site.

Soils of the kurgans						Surface soils				
#*	Object	Horizon	Depth, cm	рH_H2O_	рН_KCl_	#	Horizon	Depth, cm	рH_H2O_	рН_KCl_
4b	buried soil	Ahb	0–10	6.3	4.6	1s**	Ah	0–7	6.9	–
		E/Btb	10–20	6.4	4.4			7–14	6.3	–
		Bt1b	20–35	6.4	4.2		AhE	14–25	6.0	–
			35–44	6.4	4.2		E	25–35	5.9	–
		Bt2b	44–60	6.4	4.2		E/Bt	35–40	5.8	–
			60–80	6.4	4.3		Bt1	40–50	5.9	–
		BtCb	80–100	6.5	4.5			50–60	5.8	–
5b	burial mound		+(85–95)	6.3	4.4			60–70	5.9	–
			+(75–85)	5.9	4.0		Bt2	80–90	5.9	–
			+(65–75)	5.7	4.1			90–100	6.0	–
			+(55–65)	5.6	4.2			100–110	5.9	–
			+(40–50)	5.5	3.9	3s	Ah	0–9	6.4	5.2
			+(30–40)	5.3	3.8			9–19	6.5	4.3
			+(20–30)	5.1	3.7			19–30	6.5	4.8
			+(10–20)	5.0	3.6		E/Bt	30–46	6.6	5.0
			+(0–10)	5.4	3.7		Bt1	46–55	6.7	4.7
	buried soil	Ahb	0–5	5.6	3.8			50–60	6.7	4.5
		AhEb	5–12	5.6	3.8			60–65	6.7	4.5
		E/Btb	12–25	5.8	3.8		Bt2	80–90	6.6	4.4
		Bt1b	25–35	5.8	3.8			90–100	6.6	4.4
			35–50	5.9	3.8			100–105	6.7	4.4
			50–60	6.0	4.3	4s	Ah	0–10	6.6	6.0
			60–70	6.2	4.5		AhE	10–20	6.5	5.3
		Bt2b	70–90	6.4	4.4		E/Bt	20–30	6.3	4.7
			90–110	6.4	5.7			30–40	6.2	4.5
8b**	burial mound		+(100–120)	5.7	–			45–50	6.2	4.4
			+(75–100)	4.9	–		Bt1	50–60	6.2	4.4
			+(67–75)	5.0	–			60–70	6.2	4.3
			+(5–12)	4.9	–			70–80	6.3	4.3
			+(0–5)	4.9	–		Bt2	85–90	6.3	4.3
		Separating layer***		5.0	–			90–100	6.3	4.4
	buried soil	Ahb	0–10	5.1	–			100–105	6.4	4.4
		AEb	15–25	4.8	–	5s	Ah	0–7	6.3	5.8
		E/Btb	25–30	5.0	–			7–16	6.5	4.6
		Bt1b	30–40	5.4	–			16–30	6.6	4.9
			40–50	5.5	–		AhE	30–43	6.6	5.0
		Bt2b	50–60	5.3	–		E/Bt	43–50	6.5	4.6
			60–70	5.7	–			50–60	6.6	4.3
			70–80	5.5	–		Bt1	60–70	6.5	4.3
			80–90	5.8	–			70–80	6.5	4.2
		2Ahb	90–100	6.2	–		Bt2	80–90	6.5	4.4
9b	burial mound		+(100–110)	6.0	4.6			90–100	6.6	4.3
			+(90–100)	6.1	3.9			100–105	6.6	4.4
			+(80–90)	6.1	3.9	6s	Ah	0–15	5.7	4.2
			+(70–80)	6.1	3.7		AhE	15–36	5.7	4.1
			+(65–70)	5.7	3.7		E/Bt	36–50	5.5	3.7
			+(60–65)	5.9	3.9		Bt1	50–60	5.5	3.7
			+(50–60)	5.9	3.8			60–70	5.6	3.8
			+(40–50)	5.9	3.8			70–80	5.7	3.8
			+(30–40)	6.0	3.7			80–90	5.6	4.0
			+(20–30)	5.8	3.9			90–100	5.7	3.9
			+(10–20)	5.6	3.8			100–105	5.6	3.9
			+(0–10)	5.7	3.7	7s	Ah	0–12	6.1	5.3
		Separating layer		5.8	3.8		AhE	12–32	6.4	4.4
	buried soil		0–10	5.9	3.8		E/Bt	32–50	6.6	4.3
			10–20	5.8	3.8		Bt1	50–60	6.6	4.0
			20–30	5.9	3.7			60–70	6.6	4.0
			35–40	6.0	3.9			75–80	6.6	4.0
			40–50	6.2	3.9			80–90	6.5	4.0
			50–60	6.3	3.9			90–100	6.8	4.0
			60–70	6.3	4.1			100–105	6.7	4.0
			70–85	6.3	3.9	8s	Ah	0–8	6.3	5.7
24b	burial mound		+(95–105)	6.0	5.5			8–17	6.3	4.6
			+(85–95)	6.1	5.1			17–26	6.5	4.7
			+(75–85)	6.2	4.4		E	26–33	6.5	4.3
			+(65–75)	6.2	4.3		E/Bt	33–50	6.6	4.3
			+(60–65)	6.3	4.1		Bt1	50–60	6.6	3.9
			+(55–60)	6.3	4.1			60–70	6.0	3.9
			+(45–55)	6.0	3.7			70–80	6.2	3.8
			+(35–45)	5.8	3.6			80–90	6.1	3.9
			+(15–25)	5.9	3.7		Bt2	90–100	5.8	3.8
			+(5–15)	6.1	3.7			100–105	5.9	3.9
			+(0–5)	5.7	3.8	9s	Ah	0–10	6.2	5.4
	buried soil		0–5	5.5	4.0		AhE	15–25	6.4	4.7
			5–15	5.6	4.1		E/Bt	25–40	6.5	4.4
			15–25	5.6	3.8		Bt1	40–50	6.4	4.5
			25–35	5.7	3.9					
			35–45	5.9	3.9					
			45–50	6.0	3.9					
			50–55	6.0	4.0					
			55–65	6.1	4.0					
			65–75	6.1	4.1					
			75–85	6.0	4.3					
			85–95	6.2	4.4					
			95–105	6.2	4.5					

*Labels of boreholes or soil pits as shown in Fig. 1B.

**data from [1].

*** separating layer - a thin yellow layer excavated from the pit dug for the burial chamber, composed from material of Bt3 horizon (see also [1]).

**Fig. 5:**
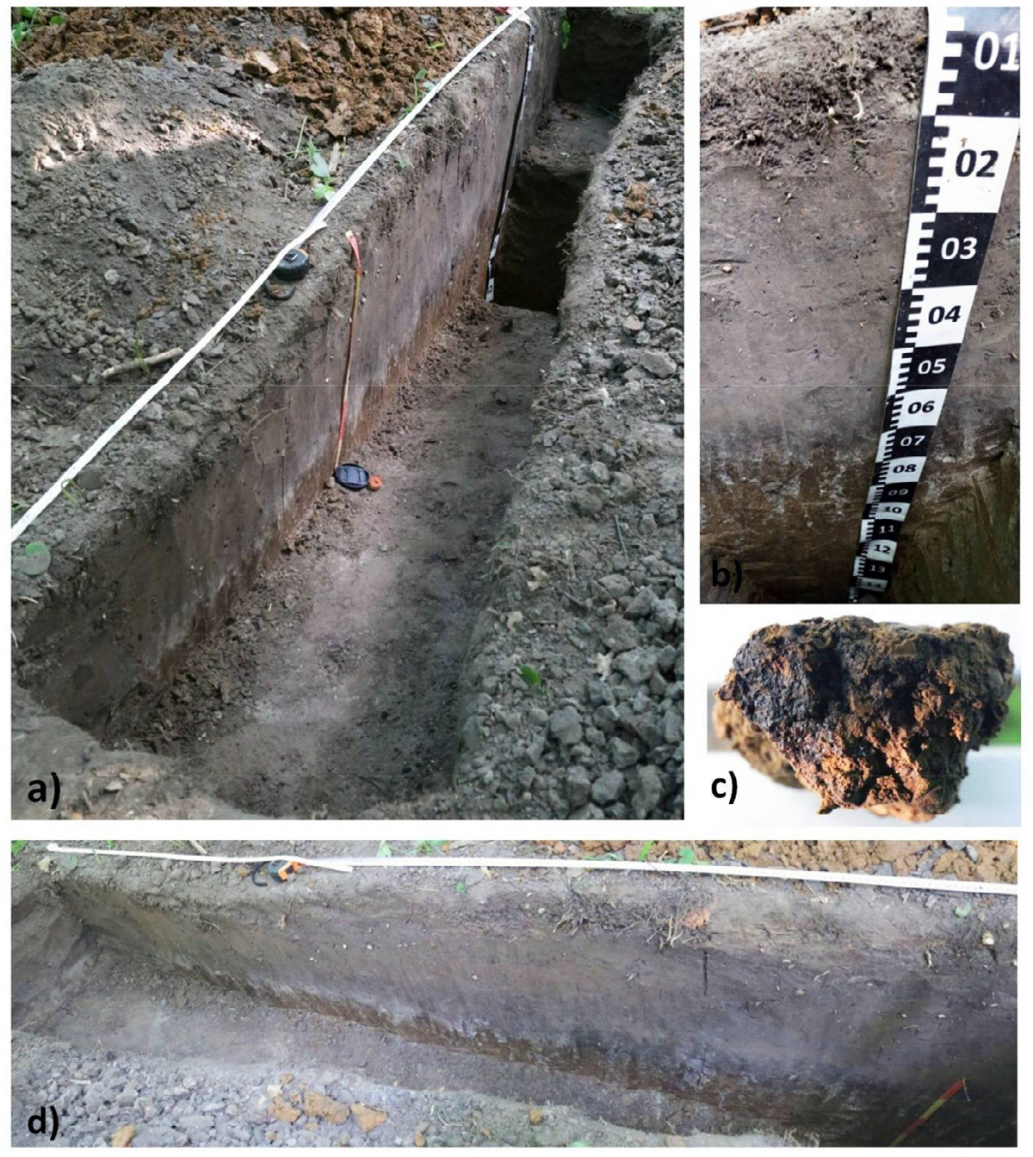
General structure of kurgan 4 and buried soil 4b (a); profile 4b of the buried soil in the central part of kurgan 4 (b); black humic coating in the BtC horizon of buried soil 4b (c); the black humus horizon disappears towards the periphery of the kurgan (d).

**Fig. 6:**
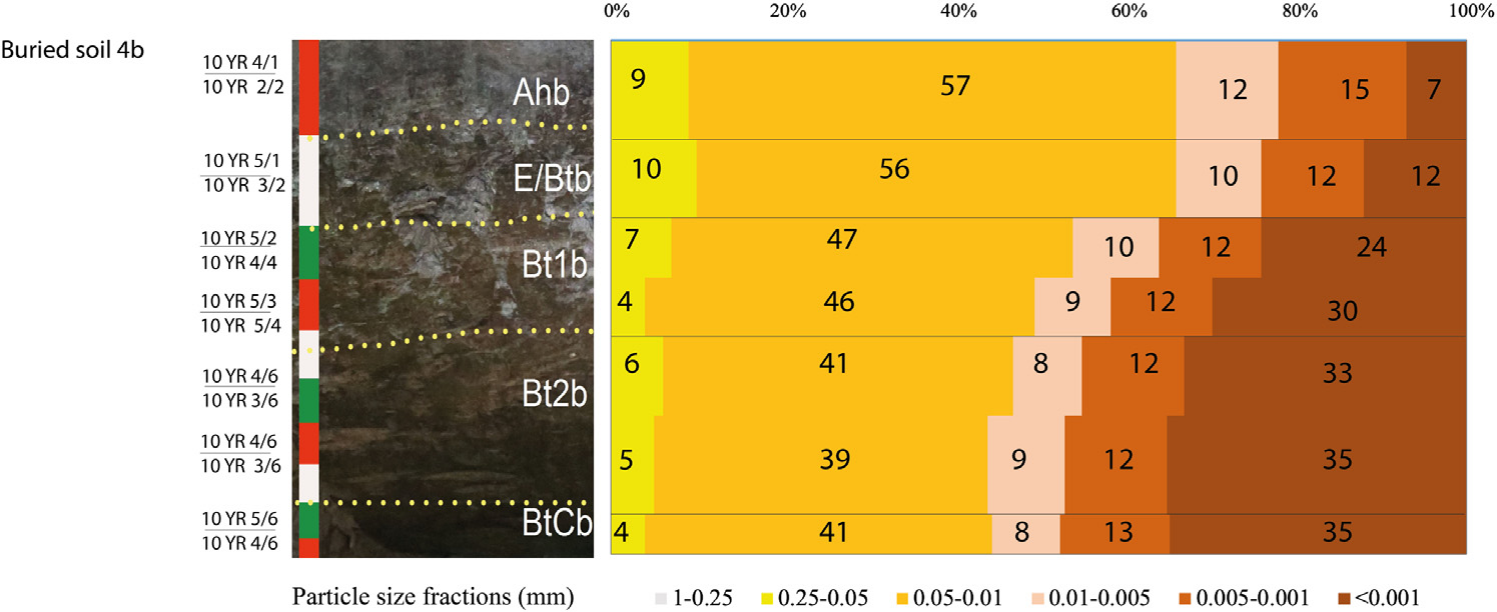
Field morphology and particle size distribution of buried soil 4b of kurgan 4 at the Tokhmeyevo kurgan cemetery.

Fig. 5 displays a transect through kurgan 4. The remains of the burial mound are only 50 cm thick. The buried soil has a dark humus horizon that disappears on the periphery of the mound (Fig. 5d). The drilling in the bottom of the soil pit discovered black humic coatings in the BtC horizon (Fig. 5c). Fig. 6 presents horizonation and particle size distribution for the buried soil 4b of kurgan 4. The soil has black humus Ahb horizon (10YR 2/2, moist) like all buried soils at the kurgan cemetery. The particle size distribution shows abrupt textural contrast similar to the buried soil 8b of kurgan 8 [1] and the surface soils (Fig. 2). The depth to Bt horizon is less in the buried soil comparing to the surface soils. It could be a result of truncation either at the time of the kurgan construction or during archaeological excavations. Table 2 contains the abundances and percentage of plant species in the pollen and spore records. These data supplement and concretize the palynological diagram published in [1].

## 2 Experimental Design, Materials and Methods

### 2.1 Dataset area and objects

The dataset area is located in the northern part of the Volga upland 20 km south of Cheboksary, Cheboksary district, Chuvash Republic, Russia, 1 km south-west from Tokhmeevo village. Soils are formed in loess sediments that are 5 – 10 m thick. The vegetation consists of the broadleaf forest with linden (*Tilia cordata*), oak (*Quercus robur*), and acer (*Acer platanoides*). The Tokhmeyevo kurgan cemetery, which is dated back to the Middle Bronze age [4], occupies a quadrangle area of ∼6000 sq.m on a slightly undulating upland slope with elevation 160 – 170 m a.s.l. (Fig. 1). The cemetery consists of 48 kurgans (Fig. 1B) with a preserved height ranging from 0.3 to 2 m. The kurgans are mostly circular with 6–20 m in diameter. The thickness of burial mounds in the sampled kurgans range from 50 to 135 cm.

Samples were collected from soil pits and boreholes beyond and within the boundaries of the Tokhmeyevo archaeological site (Fig. 1B). The sampled pedons (both surface and buried) belong to a single landscape unit since all physiographic parameters (parent material, topography, vegetation) are similar and microtopographic features are not notable. Both buried and surface soils of the dataset area are presented by Glossic Folic (Albic) Eutric Retisol (Abruptic, Loamic, Cutanic, Differentic, Ochric) [1,5]. Soils were described according to the FAO Guidelines for Soil Description [6]. Soil colour was determined using the Munsell Soil colour Charts [7].

### 2.2 Sampling procedure

Samples from the soil pits were collected from the following horizons: Ah-AhE-E-E/Bt1 for the surface soils and Ahb-AhEb-Eb-EBt-Bt1b for the buried soils. Borehole samples were taken at intervals of 10 cm (sometimes – 5 cm) from the surface down to a depth of 110 cm. The borehole samples from the kurgans and the buried soils were taken from three kurgans through the burial mound and down to a depth of 100 cm below the former day surface; kurgan 4b was sampled in a soil pit (Ahb- E/Btb-Bt1b-Bt2b- BtCb horizons). The samples from the surface soils were taken from seven soil pits deepened by boreholes. The earlier published data on soil properties of the surface and buried soil and burial mound [1] was included for comparison.

**Table 2:** Palynological spectra for the buried and surface soils (in the numerator –%, in the denominator – number of grains).

Pollen/spores by plant species	Kurgan #, horizon depth from the present surface of the kurgan mound, cm				Surface soil 1s, 0–5 cm
	8b (centre), 115–130	8b (reference section), 120–125	7b, 85–95	6b, 50–60	
**Total number of grains**	**105**	**232**	**193**	**213**	**229**
Arboreal pollen, total	63.8 67	51.3 119	43.1 83	51.2 109	80.4 184
Herbaceous pollen, total	15.3 16	18.9 44	21.2 41	15.9 34	7.4 17
Spores, total	20.9 22	29.8 69	35.7 69	32.9 70	12.2 28
**Pollen of gymnosperms, total**	**46.2** **31**	**45.3** **54**	**50.6** **42**	**44.9** **49**	**50.5** **93**
*Picea obovata Ledeb.*	–	10.1 12	3.6 3	3.7 4	1.6 3
*Pinus sibirica Du Tour*	16.4 11	6.7 8	14.5 12	11.9 12	16.8 31
*P. sylvestris* L.	13.5 9	17.6 21	22.9 19	25.7 28	29.3 54
*Abies sibirica Ledeb.*	–	–	–	0.9 1	
*Larix sibirica Ledeb.*	8.9 6	6.7 8	4.8 4	3.7 4	1.6 3
*Cupressaceae* *Juniperus communis* L.	7.4 5	4.2 5	4.8 4	–	1.2 2
**Pollen of angiosperms, total**	**53.8** **36**	**54.7** **65**	**49.4** **41**	**55.1** **60**	**49.5** **91**
*Betula sect. Albae*	17.9 12	9.4 11	14.6 12	7.3 8	11.4 21
*B. sect. Fruticosa*	6.1 4	5.9 7	4.8 4	4.6 5	4.9 9
*Alnus glutinosa (*L.*) Gaertn.*	13.4 9	7.6 9	7.2 6	12.8 14	2.2 4
*Corylus avellana* L.	6.1 4	2.5 3	2.4 2	4.6 5	5.4 10
*Tilia cordata Mill.*	–	17.6 21	10.8 9	10.3 11	19.0 35
*Quercus robur* L.	2.9 2	2.5 3	–	2.7 3	1.2 2
*Ulmus laevis Pall. et U. glabra Huds.*	–	4.2 5	3.6 3	5.5 6	2.7 5
*Acer aff. platanoides* L.	–	0.8 1	–	–	–
*Populus nigra* L.	–	4.2 5	6.0 5	–	–
*Salix caprea* L.	7.4 5	–	–	2.7 3	2.7 5
**Herbaceous pollen, total**	**42.1** **16**	**38.9** **44**	**37.3** **41**	**32.7** **34**	**37.8** **17**
*Chenopodiaceae*	5.3 2	–	3.6 4	2.8 3	4.4 2
*Poaceae*	7.9 3	6.2 7	5.4 6	4.8 5	6.7 3
*Typhaceae*	–	–	–	–	4.4 2
*Urticaceae*	–	–	2.7 3	–	–
*Papaveraceae*	2.6 1	–	–	–	–
*Lamiaceae*	7.9 3	–	–	–	4.4 2
*Liliaceae*	–	–	4.6 5	–	–
*Convallaria majalis* L.	–	–	–	6.2 7	–
*Paris quadrifolia* L.	–	–	–	5.3 6	–
*Silenaceae* *Dianthus deltoids* L.	–	0.9 1	–	3.0 3	4.4 2
*Rosaceae*	–	8.8 10	1.8 2	5.8 6	11.3 5
*Saxifragaceae*	–	0.9	–	–	–
*Onograceae* *Chamaenerium angustifolium (*L.*) Scop.*	10.5 4	1.8 2	10.2 11	5.8 6	–
*Polygonaceae* *Polygonum convolvulus* L.	–	–	2.7 3	–	–
*Iridaceae* *Gladiolus imbricatus* L.	–	4.4 5	–	–	–
*Grjssuliaceae*	–	–	–	–	–
*Liliaceae*	–	–	–	4.8 5	–
*Lamiaceae*	–	–	–	3.8 4	
*Fabaceae*	7.9 3	–	–	–	11.3 5
*Cyperaceae* *Cyperus radicaus Schkunr.*	–	2.6 3	2.7 3	1.9 2	–
**Spores, total**	**57.9** **22**	**61.1** **69**	**62.7** **69**	**67.3** **70**	**62.2** **28**
*Polypodiaceae*	28.9 11	23.0 26	23.6 26	27.9 29	26.7 12
*Lycopodiaceae* *Lycopodiella inundata (*L.*) Holub.*	15.8 6 -	12.4 14 1.8 2	17.3 19	18.3 19 1.9 2	6.7 3
*Hypolepidaceae* *Pteridium aquilinum (*L.*) Kuhn. ex Decken*	–	10.6 12	10.0 11	5.8 6	4.4 2
*Ophioglossaceae* *Botrychium virginianum (*L.*) Sw.*	5.3 2	9.7 10	6.4 7	6.7 7	8.9 4
*Sphagnum obtusum Warnst.*	7.9 3	3.5 4	5.4 6	6.7 7	11.1 5
*Selaginella aff. selaginoides (*L.*) Linc.*	–	–	–	–	2.2 1
*Bryales*	–	–	–	–	2.2 1

### 2.3 Laboratory methods

The collected 173 bulk samples were air-dried, crushed to pass through a 1 mm sieve, and analysed using potentiometric method for рН in water and 1 M KCl suspensions (a 1:2.5 soil:liquid mixture) (based on the average of two samples). The particle size distribution was performed after pre-treatment of the samples with sodium pyrophosphate [2,3] with H_2_O_2_ oxidation of organic matter. In physical fractionation, the coarse and medium sand fractions were separated from the bulk soil samples by wet sieving while fine sand and silt fractions, as well as the clay fraction, were obtained by sedimentation and siphoning, during times determined by Stokes’ law. The boundaries between particle sizes classes were defined following the Russian conventional fraction groups [2]: coarse and medium sand (1–0.25 mm), fine sand (0.25–0.05 mm), coarse silt (0.05–0.01 mm), medium silt (0.01–0.005 mm), fine silt (0.005 – 0.001 mm) and clay (<0.001 mm).

For the study of the palynological assemblage, samples were treated with HCl and KOH and centrifuged in heavy liquid (CdI + KI) and subjected to standard acetolysis [1]. In order to determine the pollen content, Lycopodium spores were added to the samples (batch No. 3862). The determination of pollen and spores was carried out under a light microscope at 400 × and 1000 × magnifications. The pollen identification was performed based on a reference collection, keys, and illustrations by [8,9]. Percentages of pollen groups were calculated from the total amount of pollen; percentages of spores were calculated referring to the total amount of pollen and spores.

## Declaration of Competing Interest

The authors declare that they have no known competing financial interests or personal relationships which have or could be perceived to have influenced the work reported in this article.
